# Long-term disability in common mental disorders in Chinese community: evidence from a five-year follow-up study

**DOI:** 10.1186/s12888-022-04382-4

**Published:** 2022-11-22

**Authors:** Yijiao Zhang, Minghui Li, Huifang Yin, Chao Ma, Zhengjing Huang, Yongping Yan, Changgui Kou, Mi Hu, Jing Wen, Shulin Chen, Cunxian Jia, Jie Yan, Hua Ding, Qiang Li, Li Yang, Yueqin Huang, Zhaorui Liu, Guangming Xu

**Affiliations:** 1 Tianjin Anding Hospital, Mental Health Center of Tianjin Medical University, No. 13, Liulin Road, Hexi District, Tianjin, 300222 China; 2 School of Education, Tianjin University, Tianjin, China; 3 Peking University Sixth Hospital (Institute of Mental Health), National Clinical Research Center for Mental Disorders & Key Laboratory of Mental Health, Ministry of Health (Peking University), No. 51 Hua Yuan Bei Road, Beijing, 100083 China; 4 National Center for Chronic and Noncommunicable Disease Control and Prevention, Beijing, China; 5 Department of Epidemiology, The Fourth Military Medical University, Xi’an, China; 6 Department of Epidemiology and Biostatistics, School of Public Health, Jilin University, Changchun, China; 7 Xiangya school of public health, Changsha, China; 8 Department of Epidemiology and Health Statistics, School of Public Health and Management at Ningxia Medical University, Yinchuan, China; 9 Department of psychological and behavior science, Zhejiang University, Hangzhou, China; 10 Department of Epidemiology, School of Public Health, Cheeloo College of Medicine, Shandong University & Shandong University Center for Suicide Prevention Research, Jinan, China; 11 School of Government, Peking University, Beijing, China; 12 Institute of Social Science Survey, Peking University, Beijing, China

**Keywords:** Common mental disorders, Long-term disability, Dysthymic disorder, Major depressive disorder, Follow-up study

## Abstract

**Background:**

Common mental disorders are general term for mental disorders with high disability rates and significant social burden. The purpose of this study was to determine the degree of long-term disability associated with common mental disorders and to interpret the relationship between common mental disorders and long-term disability.

**Methods:**

Participants in the 2013 China Mental Health Survey were followed up by telephone between April and June 2018. This study evaluated long-term disability over a five-year period using the World Health Organization’s Disability Assessment Schedule 2.0. Poisson regression was used to analyze the relationship between common mental disorders and long-term disability.

**Results:**

A total of 6269 patients were followed up by telephone. In patients with common mental disorders, the prevalence of disability ranged from 7.62% to 43.94%. The long-term disabilities were significantly associated with dysthymic disorder (DD, RR:2.40; 95% CI:1.87-3.03), major depressive disorder (MDD, RR:1.63; 95% CI:1.34-1.98), generalized anxiety disorder (GAD, RR:1.95; 95% CI:1.15-3.09), obsessive-compulsive disorder (OCD, RR:1.68; 95% CI:1.24-2.22) and alcohol use disorder (AUD, RR: 1.42; 95% CI:0.99-1.96).

**Conclusions:**

In China, common mental disorders raise the risk of long-term disability, and there is a critical need for monitoring patients with DD, MDD, GAD, OCD, and AUD. For improved quality of life and reduced disability levels, more resources need to be dedicated to mental health in the future.

## Introduction

The prevalence of mental disorders is high and contributes significantly to the burden of disease globally [1]. With the escalation of socioeconomic development and population fluctuation, the prevalence of mental disorders increases annually [2]. In 2016, mental disorders accounted for 32.4% of Years Lived with Disability (YLDs) and 13.0% of disability-adjusted life-years (DALYs) globally [3]. As a matter of fact, mental disorders contribute to the suffering of patients and burden societies and families in general [4]. Mental disorders are also prevalent in China. Approximately 15% of China’s total health expenditures and 1.1% of its gross domestic product were spent on mental disorders [5]. According to the China Mental Health Survey (CMHS) conducted in 2013, the weighted lifetime prevalence of any mental disorder excluding dementia, was 16.6% (95% CI: 13.0-20.2), and the weighted 12-month prevalence was 9.3% (95% CI: 5.4-13.3). Anxiety disorders had the highest 12-month prevalence of all mental disorders at 5.0%, followed by mood disorders at 4.1% and substance use disorders at 1.9%. Therefore, anxiety disorders, mood disorders, and substance use disorders are regarded as common mental disorders in China [6].

Functional disability resulting from mental disorders is on the rise [7]. The percentage of people with mental health disabilities increased from 2.0% in 1997-1999 to 2.7% in 2007-2009 [8]. According to the International Classification of Functioning, Disability and Health Framework (ICF), disability is defined as a personal dysfunction, including impairments, activity limitations, and participation restrictions [9–11]. There is an unmet need for effective treatment of mental disorders due to the early age of symptom onset, long tendency to relapse, and long duration [12–14]. This may result in a long-term functional disability in patients. For example, a four-year longitudinal study in the Netherlands evaluated the long-term disability of anxiety disorder patients and found that patients with any anxiety disorder had higher levels of long-term disability than healthy controls [15]. Another study of long-term disability in depressive disorder showed that patients reported more severe work disabilities on all measures than healthy controls [16].

However, the reported studies mainly focus on specific categories of mental disorders, leaving gaps in comprehensive studies of the common mental disorders. Moreover, there are few longitudinal follow-up studies in China that discuss the relationship between mental disorders and long-term disability [15, 17]. Furthermore, the current researches have small sample sizes and are slightly less representative [18, 19]. In order to monitor long-term disability, a follow-up study was conducted 5 years after the CMHS completed. Long-term disability in this study was defined as functional disability at 5 years. The purpose of our study was to investigate the extent of long-term disability in common mental disorders and to interpret the relationship between common mental disorders and long-term disability.

## Materials & methods

### Sample and procedure

#### Sample

**Fig. 1:**
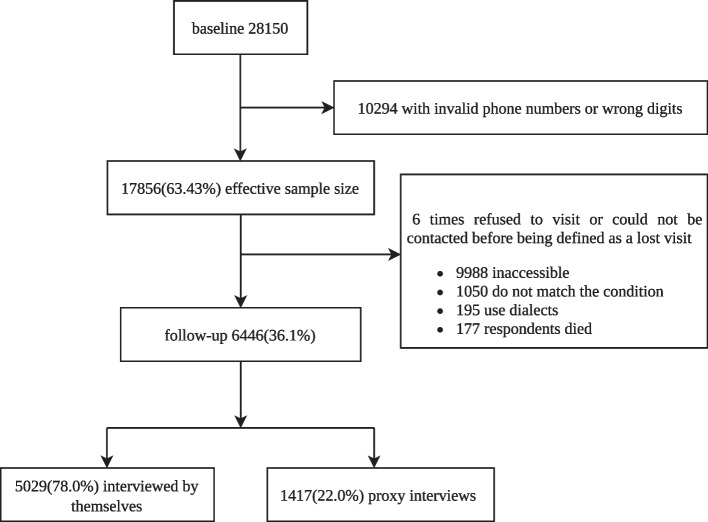
Flowchart for the participants of CMHS and follow-up study

The CMHS is the first nationally representative community survey on mental disorders and mental health services in China. The World Health Organization (WHO) Composite International Diagnostic Interview, Version 3.0 (CIDI-3.0), was used to interview 28,140 community individuals aged 18 and up in the CMHS by trained lay interviewers. Full methodologic details of the CMHS have been published [6, 20, 21]. From April to June 2018, all CMHS participants were subjected to a follow-up study using a computer assisted telephone interview (CATI) system [22, 23]. Individuals diagnosed with mood disorders, anxiety disorders, or substance-use disorders in the CMHS were included in this study, as well those without other mental disorders. Details can be found in Fig. 1.

#### Procedure

This study utilized the CATI system to interview all participants. A total of fifty interviewers attended a two-day training on the use of CATI, the study procedure, telephone interviewing skills, questionnaire content, quality control methods, and passed the final examinations. In addition, informants were interviewed if participants rejected, were unable to, or died during the follow-up period. Please refer to the published article for details on whether alternative answers are allowed [24]. The informant should be at least 18 years old and well-versed in the participant’s situation.

As well as the pre-survey preparation, various aspects of quality control were considered and designed during the formal stage of the questionnaire survey and the post-survey data verification stage to ensure the results were as accurate, usable, and representative as possible. The CATI system made it simpler to get para data during the interviews. Daily data checks were performed on all interviews to detect systematic errors and their distribution patterns, focusing on non-response, stoppage of interviews, and contact attempt times. Audio checks were performed on around 10% of respondents to uncover interviewer problems such as data entry errors, erroneous informant information, inadequate probing, and irregular behaviors while asking a question. Monitoring checks were performed on at least three telephone calls by each interviewer to ensure that the interviewers acted appropriately during the interviews. Further quality control details were published [24].

The study was authorized by the Ethical Committee of Tianjin Anding Hospital and the Sixth Hospital of Peking University. Prior to their involvement in the study, all participants and informants were given oral informed consents, and the consent procedure was recorded to audio files.

### Materials

#### Diagnose of common mental disorders

Past 12-month diagnoses of mental disorders were assessed with the CIDI-3.0, which classifies diagnoses according to the American Psychiatric Association’s Diagnostic and Statistical Manual, Edition IV (DSM-IV) criteria. The CIDI is a fully-structured diagnostic interview conducted by trained lay interviewers [22]. The CIDI has been proven to be valid and is used in China [6] and many other countries for epidemiological surveys [25].

The diagnosis of common mental disorders in this study was assessed according to the criteria of DSM-IV [21], and divided into three categories: (1) anxiety disorders [panic disorder (PD), specific phobia (SP), social phobia (SO), obsessive-compulsive disorder (OCD), generalized anxiety disorder (GAD)]; (2) mood disorders [major depressive disorder (MDD), dysthymic disorder (DD), and bipolar disorder (BD)]; and (3) substance-use disorders (alcohol use disorder (AUD)).

#### Assessment for long-term disability

At both the baseline and this 5-year follow-up, the functional disability of participant was assessed using the revised version of the WHO Disability Assessment Schedule (WHODAS-2.0), based on the International Classification of Functions [26]. The internal consistency and validity of the World Mental Health Survey WHODAS-2.0 have been demonstrated [27]. The Chinese version of the WHODAS-2.0 has good internal consistency reliability and empirical validity, and the Cronbach’s α was 0.73-0.99 [28]. WHODAS-2.0 includes six domains: 1) cognition – understanding and communicating; 2) mobility – moving and getting around; 3) self-care – attending to one’s hygiene, dressing, eating and staying alone; 4) getting along – interacting with other people; 5) life activities – domestic responsibilities, leisure, work and school; 6) participation – joining in community activities, participating in society.

Based on the distributional features of the instrument, the global disability score was dichotomized at the 90th percentile to reflect the presence or absence of considerable disability, as recommended by Von Korff et al. [27].

#### Contact information

Baseline and follow-up contact information was collected for tracking purposes, including the address, plans to move to a new home, and contact information for participants and informants.

#### Covariates

Covariates were used to adjust for possible differences in disability experience, including sex, age, participant/proxy, residence area, marital status, and education. The WHODAS category at baseline was also adjusted as a covariate, since disability at baseline is related to long-term disability.

### Statistical analysis

Sampling design weights, non-response adjustment weights, post-stratification adjustment, and trimming of the weights were used when calculating prevalence and the risk ratios (RRs). The methods of weighting were similar as the way to those used in the CMHS, which have been published elsewhere [21]. This study differed from the CMHS in terms of weights primarily because it used a different method for calculating non-response weights, which was also adjusted according to the diagnosis of mental disorder [24].

Frequencies and chi-square (χ^2^) tests were used for descriptive analysis and comparisons of rates in different categories. The RRs for common mental disorders-related disability were estimated using Poisson regression. Throughout the study, a significance threshold of 0.05 was applied. SAS 9.4 and R 4.0.2 were used for statistical analysis.

## Results

### General sociodemographic characteristics

During the follow-up study, interviewers attempted to contact participants who had valid telephone numbers for themselves or their informants. The final weighted sample was 6269. Overall, 5435 (86.3%) participants completed the questionnaires themselves, while 864 (13.7%) were informants who completed proxy interviews. A total of 51.01% (*n* = 3198) of the 6269 participants interviewed were males, while 48.99% (*n* = 3071) were females. As for education levels, illiteracy or below primary school was the highest (19.94%, *n* = 1246), whereas university or above was the lowest (9.95%, *n* = 622). In terms of marital status, 5406 married (86.25%) and 862 single (13.75%) participants were present. Table 1 presents the general sociodemographic characteristics of the sample.

**Table 1:** General sociodemographic characteristics

Variables	Categories	Weighted Frequency *n* (%)	95% CI (%)
Age in years	18-34	2173 (34.74)	31.41-38.07
	35-49	2036 (32.54)	30.11-34.97
	50-64	1447 (23.12)	20.99-25.26
	>65	601 (9.60)	8.23-10.97
Sex	Male	3198 (51.01)	48.76-53.27
	Female	3071 (48.99)	46.73-51.24
Education	Literate or below primary school	1246 (19.94)	17.60-22.28
	Primary school	1312 (21.00)	18.72-23.28
	Junior high school	2160 (34.56)	31.98-37.15
	Senior high school	909 (14.55)	12.63-16.48
	College or university and above	622 (9.95)	7.55-12.34
Marital status	Married	5406 (86.25)	84.07-88.43
	Separated/Divorced	271 (4.32)	3.50-5.15
	Never married	591 (9.43)	7.36-11.50
Employment status	In work	2835 (45.36)	42.08-48.63
	Not in work	3415 (54.64)	51.37-57.92
Residence area	Urban	3660 (58.38)	51.76-65.01
	Rural	2609 (41.62)	34.99-48.24
WHODAS category at baseline	With disability	567 (9.04)	7.64-10.45
	No disability	5702 (90.96)	89.56-92.36

### 30-day prevalence of disability of common mental disorders

In this study, 15.41% (86/559) of patients with common mental disorders met the disability criteria (95% CI: 9.67-21.15). In China, mood disorders were the leading cause of disability. 33.27% (58/175) of participants with any mood disorder reported disability (95% CI: 19.94-46.60). The disability rates for MDD and DD were 32.17 and 43.94%, respectively. This rate was 13.74% (35/256, 95% CI: 8.09-19.39) among those with any anxiety disorder, with GAD (29.63%) being the most prevalent. 7.62% (11/139) of those with any substance-use disorder (95% CI: 0.99-1.75). Table 2 displays the specific 30-day prevalence of disability.

**Table 2:** 30-day prevalence of disability

	Weighted N	Weighted n	(%)	95% CI
30-day Disability (90th percentile)	559	86	15.41	9.67-21.15
Mood disorders				
Any mood disorder	175	58	33.27	19.94-46.60
Major depressive disorder	131	42	32.17	17.26-47.09
Dysthymic disorder	55	24	43.94	19.53-68.34
Bipolar disorders	22	5	21.33	6.16-36.50
Anxiety disorders				
Any anxiety disorder	256	35	13.74	8.09-19.39
Panic attack	25	4	16.43	0.00-45.06
Specific phobia	73	12	17.67	6.39-51.92
Obsessive compulsive disorder	114	16	13.77	4.87-22.68
Generalized anxiety disorder	18	5	29.63	0.00-60.64
Substance-use disorders				
Any substance use disorder	139	11	7.62	2.22-13.01
alcohol use disorder	130	10	7.90	2.14-13.65

### Association between disability and common mental disorders

The results indicate that after adjusting for sex, age, participant/proxy, residence area, marital status, education, and the WHODAS category at baseline, the relationship between common mental disorders and long-term disability was: the association between any mood disorder and long-term disability was significant, with participants with any mood disorder 1.94 (95% CI: 1.65-2.27) times more likely to report disability than participants without mood disorders. The relationship between any anxiety disorder and any substance use disorder and long-term disability was not significant. DD, MDD, GAD, OCD, and AUD were statistically significantly associated with long-term disability. The most disabling condition was DD (RR: 2.40; 95% CI:1.87-3.03). Table 3 shows the relationship between disability and common mental disorders.

**Table 3:** Association of common mental disorders with disability

	χ^2^	P	Adjusted RR (95%CI) ^a^
Mood disorders			
Any mood disorder	66.76	< 0.0001	1.94 (1.65-2.27) ^***^
Major depressive disorder	24.73	< 0.0001	1.63 (1.34-1.98) ^***^
Dysthymic disorder	51.01	< 0.0001	2.40 (1.87-3.03) ^***^
Bipolar disorders	3.57	0.0587	1.63 (0.94-2.59)
Anxiety disorders			
Any anxiety disorder	2.98	0.0843	1.18 (0.97-1.42)
Panic disorder	0.24	0.6222	0.88 (0.50-1.41)
Specific phobia	0.54	0.4622	1.10 (0.85-1.40)
Obsessive compulsive disorder	12.45	0.0004	1.68 (1.24-2.22) ^***^
Generalized anxiety disorder	7.05	0.0079	1.95 (1.15-3.09) ^**^
Substance-use disorders			
Any substance use disorder	3.82	0.0507	1.33 (0.99-1.75)
alcohol use disorder	3.98	0.0462	1.42 (0.99-1.96) ^*^

^a^ Estimates are based on the Poisson regression models controlling for sex, age, participant/proxy, residence area, marital status, education, WHODAS category at baseline

* *P* < 0.05

** *P* < 0.01

*** *P* < 0.001

## Discussion

This is the first study to explore long-term disability among Chinese people suffering from common mental disorders in a comprehensive way. In the CMHS 5-year follow-up study, the weighted disability rate for patients with mental disorders increased from 9% to 15.41%. The main finding of this study was that any mood disorders and long-term disability were significantly correlated, with DD and MDD being particularly influential. In addition, GAD, OCD, and AUD were all strongly associated with long-term disability. After 5 years, approximately 7.62 to 43.94% of patients with common mental disorders became disabled. The findings of this study may be valuable for policymakers in determining whether the current mental health care system can meet demand, rationalize resource allocation, and improve the long-term prognosis of people with mental disorders.

According to the present study, DD was the most disabling condition. Patients with DD were 2.4 times more likely to be disabled than health controls. This is similar to previous research. Sandhu et al. pointed out that the chronic nature of DD may result in greater functional impairment than acute depression [29]. Bernhard et al. noted that DD often co-occurs with other more severe medical comorbidities, resulting in higher long-term disability than MDD [30, 31]. Hellerstein et al. observed that DD sufferers had difficulty working full-time, which increased their susceptibility to long-term disability in terms of function [32]. In addition, a diagnosis of DD, requires 2 years of symptoms (DSM-IV), whereas a diagnosis of MDD requires only 2 weeks of symptoms. No distinct episodes and long duration are the two defining characteristics of DD [31, 33]. Moreover, the delayed treatment rate for DD is high, some people may seek help decades after the onset of symptoms [34–36], but DD is treatable to alleviate symptoms [37]. DD is a recurrent long-term depressive disorder with ill-defined episodes, high rates of delayed treatment, and high rates of co-morbidity, and these factors may further contribute to long-term disability [30, 31, 38, 39]. Similar to the findings of previous research, MDD was also associated with long-term disability [40–42]. MDD is associated with substantial disability across many domains of life [43], affecting patients’ ability to fulfill family roles, work, and participate in society at levels similar to or worse than those reported in chronic somatic diseases [44].

In our study, BD did not disclose any significant outcomes [45–48]. This finding was not in line with previous research. According to Antunes et al., there existed an association between BD and disability, but their study was cross-sectional and cannot describe the long-term disability of patients [45]. Martinez-Aran et al. concluded that BD was associated with functional disability, however, they used a clinical population as opposed to ours [48]. According to the report [49], the number of patients with BD in community was lower than in institution due to the fact that patients with BD are more likely to require hospitalization. As a result of the small number of patients with BD that participated in this study, we may have lacked statistical power to obtain significant effects [6].

In addition to mood disorders, GAD, OCD and AUD showed statistically significant associations with disability. This was similar to previous studies. These disorders are associated with long-term disability more often than not due to the symptomatic features of the disease itself, and they rarely receive formal systemic treatment [15, 50–57]. In this study, people with GAD, OCD, MDD, or AUD were nearly twice as likely to report a disability as people without these disorders. Therefore, while increasing public medical and health investment, the guarantee of long-term medical and health services for patients with mental disorders should be the focus issue.

## Limitations

There are several limitations in the research. First, long-term disability was assessed only 30 days prior to follow-up, whereas mental disorders were 12-month based. For episodic conditions, the past month of disability may not include the period of the disorder. A 12-month diagnosis also allowed for the inclusion of remitted disorders that might have residual adverse effects on disability [56]. Second, the number of certain diseases patients followed up in this study was relatively small, which may be biased to some extent in the representativeness of the sample. Nevertheless, for the follow-up study, we adopted complex weighting and non-response weighting, as well as multiple regression analysis to adjust for bias due to the different distributions of participants who were retained and those who were lost-to-follow-up. Third, since the skip strategy used in the follow-up process and the use of informants as responses if participants were unable to respond individually, it is possible that participant disability was underestimated. Even so, considering the difficulties of conducting an interview with CATI design, such tactics might be the most effective way to increase the response rate.

## Conclusions

In the Chinese community, patients with common mental disorders were at an increased risk of long-term functional disability. To reduce the risk of long-term disability, it is necessary to pay additional attention to the long-term functional status of patients with DD, MDD, GAD, OCD, and AUD. Furthermore, in the future, mental health resources should be more inclined toward these disorders in order to improve their quality of life and reduce the disability rate.

## Data Availability

The data that support the findings of this study are available from the corresponding authors on request.

## Abbreviations

YLDs: Years Lived with Disability
DALYs: Disability-Adjusted Life Years
CMHS: China Mental Health Survey
WHO: World Health Organization
CIDI-3.0: the Composite International Diagnostic Interview-3.0
CATI: Computer assisted telephone interview
DSM-IV: The Diagnostic and Statistical Manual of Mental Disorders, Fourth Edition
PD: Panic disorder
SP: Specific phobia
SO: Social phobia
OCD: Obsessive compulsive disorder
GAD: General anxiety disorder
MDD: Major depressive disorder
DD: Dysthymic disorder
BD: Bipolar disorder
AUD: Alcohol use disorder
WHODAS-2.0: World Health Organization’s Disability Assessment Schedule II Declarations
RRs: Risk ratios

## References

[1] Vigo D, Thornicroft G, Atun R (2016). Estimating the true global burden of mental illness. Lancet Psychiatry.

[2] Santomauro DF, Herrera AMM, Shadid J, Zheng P, Ashbaugh C, Pigott DM, Abbafati C, Adolph C, Amlag JO, Aravkin AY (2021). Global prevalence and burden of depressive and anxiety disorders in 204 countries and territories in 2020 due to the COVID-19 pandemic. Lancet.

[3] Hay SI, Abajobir AA, Abate KH, Abbafati C, Abbas KM, Abd-Allah F, Abdulle AM, Abebo TA, Abera SF, Aboyans V (2017). Global, regional, and national disability-adjusted life-years (DALYs) for 333 diseases and injuries and healthy life expectancy (HALE) for 195 countries and territories, 1990-2016: a systematic analysis for the global burden of disease study 2016. Lancet.

[4] Ferrari AJ, Santomauro DF, Herrera AMM, Shadid J, Ashbaugh C, Erskine HE, Charlson FJ, Degenhardt L, Scott JG, McGrath JJ (2022). Global, regional, and national burden of 12 mental disorders in 204 countries and territories, 1990-2019: a systematic analysis for the global burden of disease study 2019. Lancet Psychiatry.

[5] Xu JF, Wang J, Wimo A, Qiu CX. The economic burden of mental disorders in China, 2005-2013: implications for health policy. Bmc Psychiatry. 2016;16. 10.1186/s12888-016-0839-0 PMC4864926 27169936

[6] Huang YQ, Wang Y, Wang H, Liu ZR, Yu X, Yan J, Yu YQ, Kou CG, Xu XF, Lu J (2019). Prevalence of mental disorders in China: a cross-sectional epidemiological study. Lancet Psychiatry.

[7] Richter D, Wall A, Bruen A, Whittington R (2019). Is the global prevalence rate of adult mental illness increasing? Systematic review and meta-analysis. Acta Psychiatr Scand.

[8] Mojtabai R (2011). National trends in mental health disability, 1997-2009. Am J Public Health.

[9] Goodier J. A dictionary of epidemiology (6th edition). Ref Rev. 2015;29(4):34.

[10] Ma EPM, Worrall L, Threats TT (2007). The international classification of functioning, disability and health (ICF) in clinical practice. Semin Speech Lang.

[11] ÜstÜn TB, Ebrary I (2010). Measuring health and disability: manual for WHO Disability Assessment Schedule (WHODAS 2.0).

[12] Ormel J, Petukhova M, Chatterji S, Aguilar-Gaxiola S, Alonso J, Angermeyer MC, Bromet EJ, Burger H, Demyttenaere K, de Girolamo G (2008). Disability and treatment of specific mental and physical disorders across the world. Br J Psychiatry.

[13] Alonso J, Angermeyer MC, Bernert S, Bruffaerts R, Brugha TS, Bryson H, de Girolamo G, de Graaf R, Demyttenaere K, Gasquet I (2004). Disability and quality of life impact of mental disorders in Europe: results from the European study of the epidemiology of mental disorders (ESEMeD) project. Acta Psychiatr Scand.

[14] Demyttenaere K, Bruffaerts R, Posada-Villa J, Gasquet I, Kovess V, Lepine JP, Angermeyer MC, Bernert S, de Girolamo G, Morosini P (2004). Prevalence, severity, and unmet need for treatment of mental disorders in the World Health Organization world mental health surveys. JAMA-J Am Med Assoc.

[15] Hendriks SM, Spijker J, Licht CMM, Hardeveld F, de Graaf R, Batelaan NM, et al. Long-term disability in anxiety disorders. Bmc Psychiatry. 2016;16. 10.1186/s12888-016-0946-y PMC4950589 27431392

[16] Hendriks SM, Spijker J, Licht CMM, Hardevel F, de Graaf R, Batelaan NM, Penninx B, Beekman ATF (2015). Long-term work disability and absenteeism in anxiety and depressive disorders. J Affect Disord.

[17] Frey BN, Vigod S, Cardoso TD, Librenza-Garcia D, Favotto L, Perez R, et al. The early burden of disability in individuals with mood and other common mental disorders in Ontario, Canada. JAMA Netw Open. 2020;3(10). 10.1001/jamanetworkopen.2020.20213 PMC7588941 33104205

[18] Melzer D, Fryers T, Jenkins R, Brugha T, McWilliams B (2003). Social position and the common mental disorders with disability - estimates from the National Psychiatric Survey of Great Britain. Soc Psychiatry Psychiatr Epidemiol.

[19] Soderberg M, Stattin M, Robroek SJW, Burdorf A, Jarvholm B (2021). Industry mobility and disability benefits in heavy manual jobs: a cohort study of Swedish construction workers. Scand J Work Environ Health.

[20] Huang YQ, Liu ZR, Wang H, Guan X, Chen HG, Ma C, Li Q, Yan J, Yu YQ, Kou CG (2016). The China mental health survey (CMHS): I. background, aims and measures. Soc Psychiatry Psychiatr Epidemiol.

[21] Liu ZR, Huang YQ, Lv P, Zhang TT, Wang H, Li Q, Yan J, Yu YQ, Kou CG, Xu XF (2016). The China mental health survey: II. Design and field procedures. Soc Psychiatry Psychiatr Epidemiol.

[22] Kessler RC, Ustun TB (2004). The world mental health (WMH) survey initiative version of the World Health Organization (WHO) composite international diagnostic interview (CIDI). Int J Methods Psychiatr Res.

[23] Lu J, Huang YQ, Liu ZR, Cao XL (2015). Validity of Chinese version of the composite international diagnostic Interview-3.0 in psychiatric settings. Chin Med J.

[24] Liu ZR, Li PJ, Yin HF, Li MH, Yan J, Ma C, et al. Future trends in disability and its determinants among Chinese community patients with anxiety disorders: evidence from a 5-year follow-up study. Front Psychiatry. 2021;12. 10.3389/fpsyt.2021.777236 PMC8695844 34955923

[25] Andrews G, Henderson S, Hall W (2001). Prevalence, comorbidity, disability and service utilisation - overview of the Australian National Mental Health Survey. Br J Psychiatry.

[26] Koopmans AB, van Hoeken D, Clarke DE, Vinkers DJ, van Harten PN, Hoek HW. Proxy WHO disability assessment schedule 2.0 is clinically useful for assessing psychosocial functioning in severe mental illness. Front Psychiatry. 2020;11. 10.3389/fpsyt.2020.00303 PMC7174765 32351419

[27] Von Korff M, Crane PK, Alonso J, Vilagut G, Angermeyer MC, Bruffaerts R, de Girolamo G, Gureje O, de Graaf R, Huang Y (2008). Modified WHODAS-II provides valid measure of global disability but filter items increased skewness. J Clin Epidemiol.

[28] Chiu TY, Yen CF, Chou CH, Lin JD, Hwang AW, Liao HF, Chi WC (2014). Development of traditional Chinese version of World Health Organization disability assessment schedule 2.0 36-item (WHODAS 2.0) in Taiwan: validity and reliability analyses. Res Dev Disabil.

[29] Sandhu RS, Ghosh S, Dellenbaugh T (2016). Association between dysthymic disorder and disability, with religiosity as moderator. Act Nerv Super.

[30] Sansone RA, Sansone LA (2009). Dysthymic disorder: forlorn and overlooked?. Psychiatry (Edgmont).

[31] Baune BT, Caniato RN, Arolt V, Berger K (2009). The effects of dysthymic disorder on health-related quality of life and disability days in persons with comorbid medical conditions in the general population. Psychother Psychosom.

[32] Hellerstein DJ, Agosti V, Bosi M, Black SR (2010). Impairment in psychosocial functioning associated with dysthymic disorder in the NESARC study. J Affect Disord.

[33] Uher R (2014). MUDr: persistent depressive disorder, dysthymia, and chronic depression: update on diagnosis, Treatment. The Psychiatric times.

[34] Hellerstein DJ (2015). Dysthymic disorder (persistent depressive disorder).

[35] ten Have M, de Graaf R, van Dorsselaer S, Beekman A (2013). Lifetime treatment contact and delay in treatment seeking after first onset of a mental disorder. Psychiatr Serv.

[36] Vaingankar JA, Rekhi G, Subramaniam M, Abdin E, Chong SA (2013). Age of onset of life-time mental disorders and treatment contact. Soc Psychiatry Psychiatr Epidemiol.

[37] John W, Williams J, Barrett J, Oxman T, Frank E, Katon W, et al. Treatment of dysthymia and minor depression in primary care a randomized controlled trial in older adults. Treatment Comparisons of Dysthymia. 2000;284. 10.1001/jama.284.12.1519 11000645

[38] Niculescu Iii AB, Akiskal HS (2001). Proposed endophenotypes of dysthymia: evolutionary, clinical and pharmacogenomic considerations. Mol Psychiatry.

[39] Ishizaki J, Mimura M. Dysthymia and apathy: diagnosis and treatment. Depress Res Treat. 2011;2011:–893905. 10.1155/2011/893905 PMC3130974 21747995

[40] Kessler RC, Frank RG (1997). The impact of psychiatric disorders on work loss days. Psychol Med.

[41] Bijl RV, Ravelli A (2000). Current and residual functional disability associated with psychopathology: findings from the Netherlands mental health survey and incidence study (NEMESIS). Psychol Med.

[42] Iancu SC, Wong YM, Rhebergen D, van Balkom A, Batelaan NM (2020). Long-term disability in major depressive disorder: a 6-year follow-up study. Psychol Med.

[43] Kamenov K, Cabello M, Caballero FF, Cieza A, Sabariego C, Raggi A, et al. Factors related to social support in neurological and mental disorders. PLoS One. 2016;11(2). 10.1371/journal.pone.0149356 PMC4764676 26900847

[44] Meuleman E, Comhaire F. Organ failure and common disease of the ageing male. Andrology for the Clinician. 2006:251–4.

[45] Antunes A, Frasquilho D, Azeredo-Lopes S, Neto D, Silva M, Cardoso G, Caldas-de-Almeida JM (2018). Disability and common mental disorders: results from the world mental health survey initiative Portugal. Eur Psychiatry.

[46] Sanchez-Moreno J, Martinez-Aran A, Tabares-Seisdedos R, Torrent C, Vieta E, Ayuso-Mateos JL (2009). Functioning and disability in bipolar disorder: an extensive review. Psychother Psychosom.

[47] Kupfer DJ (2005). The increasing medical burden in bipolar disorder. JAMA-J Am Med Assoc.

[48] Martinez-Aran A, Vieta E, Reinares M, Colom F, Torrent C, Sanchez-Moreno J, Benabarre A, Goikolea JM, Comes M, Salamero M (2004). Cognitive function across manic or hypomanic, depressed, and euthymic states in bipolar disorder. Am J Psychiatry.

[49] Goncalves-Pinho M, Freitas A, von Doellinger O, Ribeiro JP (2022). Bipolar disorder related hospitalizations - a descriptive Nationwide study using a big data approach. Psychiatr Q.

[50] Kaila-Kangas L, Kivekas T, Laitinen J, Koskinen A, Harkanen T, Hirvonen L, Leino-Arjas P (2015). Abstinence and current or former alcohol use as predictors of disability retirement in Finland. Scand J Public Health.

[51] Marr NS, Zainal NH, Newman MG (2022). Focus on and venting of negative emotion mediates the 18-year bi-directional relations between major depressive disorder and generalized anxiety disorder diagnoses. J Affect Disord.

[52] Visvalingam S, Crone C, Street S, Oar EL, Gilchrist P, Norberg MM (2022). The causes and consequences of shame in obsessive-compulsive disorder. Behav Res Ther.

[53] Mancebo MC, Greenberg B, Grant JE, Pinto A, Eisen JL, Dyck I, Rasmussen SA (2008). Correlates of occupational disability in a clinical sample of obsessive-compulsive disorder. Compr Psychiatry.

[54] Cheng HG, Deng F, Xiong W, Phillips MR (2015). Prevalence of alcohol use disorders in mainland China: a systematic review. Addiction.

[55] Huang H, Chen HX, Dong HX, Ning K, Zhang RL, Sun W, Li B, Jiang HF, Wang WZ, Du J (2017). Prevalence, correlates and treatment status of alcohol use disorders in psychiatric patients in China. Gen Hosp Psychiatry.

[56] Alonso J, Petukhova M, Vilagut G, Chatterji S, Heeringa S, Ustun TB, Alhamzawi AO, Viana MC, Angermeyer M, Bromet E (2011). Days out of role due to common physical and mental conditions: results from the WHO world mental health surveys. Mol Psychiatry.

[57] Lazarov A, Oren E, Liberman N, Gur S, Hermesh H, Dar R (2022). Attenuated access to emotions in obsessive-compulsive disorder. Behav Therapy.

